# Glycometabolism-related gene signature of hepatocellular carcinoma predicts prognosis and guides immunotherapy

**DOI:** 10.3389/fcell.2022.940551

**Published:** 2022-07-22

**Authors:** Lihua Yu, Xiaoli Liu, Xinhui Wang, Huiwen Yan, Qing Pu, Yuqing Xie, Juan Du, Zhiyun Yang

**Affiliations:** ^1^ Center of Integrative Medicine, Beijing Ditan Hospital, Capital Medical University, Beijing, China; ^2^ First Clinical Medical College, Beijing University of Chinese Medicine, Beijing, China; ^3^ Beijing Key Laboratory of Emerging Infectious Diseases, Institute of Infectious Diseases, Beijing Ditan Hospital, Capital Medical University, Beijing, China; ^4^ Beijing Institute of Infectious Diseases, Beijing, China; ^5^ National Center for Infectious Diseases, Beijing Ditan Hospital, Capital Medical University, Beijing, China

**Keywords:** hepatocellular carcinoma, glycometabolism, prognosis, immunotherapy, nomogram

## Abstract

Hepatocellular carcinoma (HCC) is a severe cancer endangering human health. We constructed a novel glycometabolism-related risk score to predict prognosis and immunotherapy strategies in HCC patients. The HCC data sets were obtained from the Cancer Genome Atlas (TCGA) and the Gene Expression Omnibus (GEO) database, and the glycometabolism-related gene sets were obtained from the Molecular Signature Database. The least absolute contraction and selection operator (LASSO) regression model was used to construct a risk score based on glycometabolism-related genes. A simple visual nomogram model with clinical indicators was constructed and its effectiveness in calibration, accuracy, and clinical value was evaluated. We also explored the correlation between glycometabolism-related risk scores and molecular pathways, immune cells, and functions. Patients in the low-risk group responded better to anti-CTLA-4 immune checkpoint treatment and benefited from immune checkpoint inhibitor (ICI) therapy. The study found that glycometabolism-related risk score can effectively distinguish the prognosis, molecular and immune-related characteristics of HCC patients, and may provide a new strategy for individualized treatment.

## Introduction

According to the latest global cancer report, hepatocellular carcinoma (HCC) is the sixth most common cancer worldwide and has the third-highest mortality rate (Sung et al., 2021). The World Health Organization (WHO) has projected that the incidence rate of HCC will exceed one million by 2025 (International Agency for Research on Cancer, 2020). China is one of the major countries with a high incidence and mortality of HCC, accounting for approximately half of the total cases worldwide (Feng et al., 2019; Wang et al., 2014). Currently, the effectiveness of various treatments for HCC is unsatisfactory (Forner et al., 2018). Therefore, there is an urgent need to clarify the specific molecular mechanisms related to HCC for targeted therapy, which is expected to improve the survival rate of patients with HCC, delay tumor progression, and improve the quality of life of patients. Immunotherapy, particularly immune checkpoint inhibitor (ICI) therapy, which has achieved significant clinical breakthroughs, is still faced with challenges, such as low response rate and poor efficacy in some patients (Darvin et al., 2018; Li et al., 2019). The relationship between metabolic restriction and immunity has gradually become a topic of interest and has received considerable attention (DePeaux and Delgoffe, 2021).

In the tumor microenvironment (TME), glycometabolism is the main metabolic pathway of tumor cells and immune cells (Bose and Le, 2018). Glycometabolism reprogramming is one of the main features of the TME, and tumor cells upregulate glycolytic pathways, undergo tumor escape, and inhibit immune effector cell function until exhaustion occurs (Shevchenko and Bazhin, 2018). In the tumor microenvironment, the IL-10-fc fusion protein has been found to enhance the expansion and effector function of depleted CD8^+^ T lymphocytes by promoting the oxidative phosphorylation pathway in glycometabolism (Guo et al., 2021). The NF-E2-related factor 2 (Nrf2) antioxidant pathway was found to restore the metabolism and function of natural killer (NK) cells in human ovarian cancer (Poznanski et al., 2021). Another study found that programmed death-ligand 1 (PD-L1) signaling activates the Akt/mTOR pathway to promote glycolysis in tumor cells. Therapeutic blockade of PD-L1 inhibits tumor progression by triggering the internalization of PD-L1 and reducing the rate of glycolysis (Chang et al., 2015). Based on this, active efforts are being made to identify therapeutic targets for glycometabolism to enhance the effector function of exhausted immune cells and improve responsiveness to ICI therapy. A few biomarkers of glycometabolism in liver cancer can be used to predict patient prognosis. Identifying potential prognostic markers associated with therapy can enable personalized metabolic immunotherapy in patients with HCC.

**FIGURE 1:**
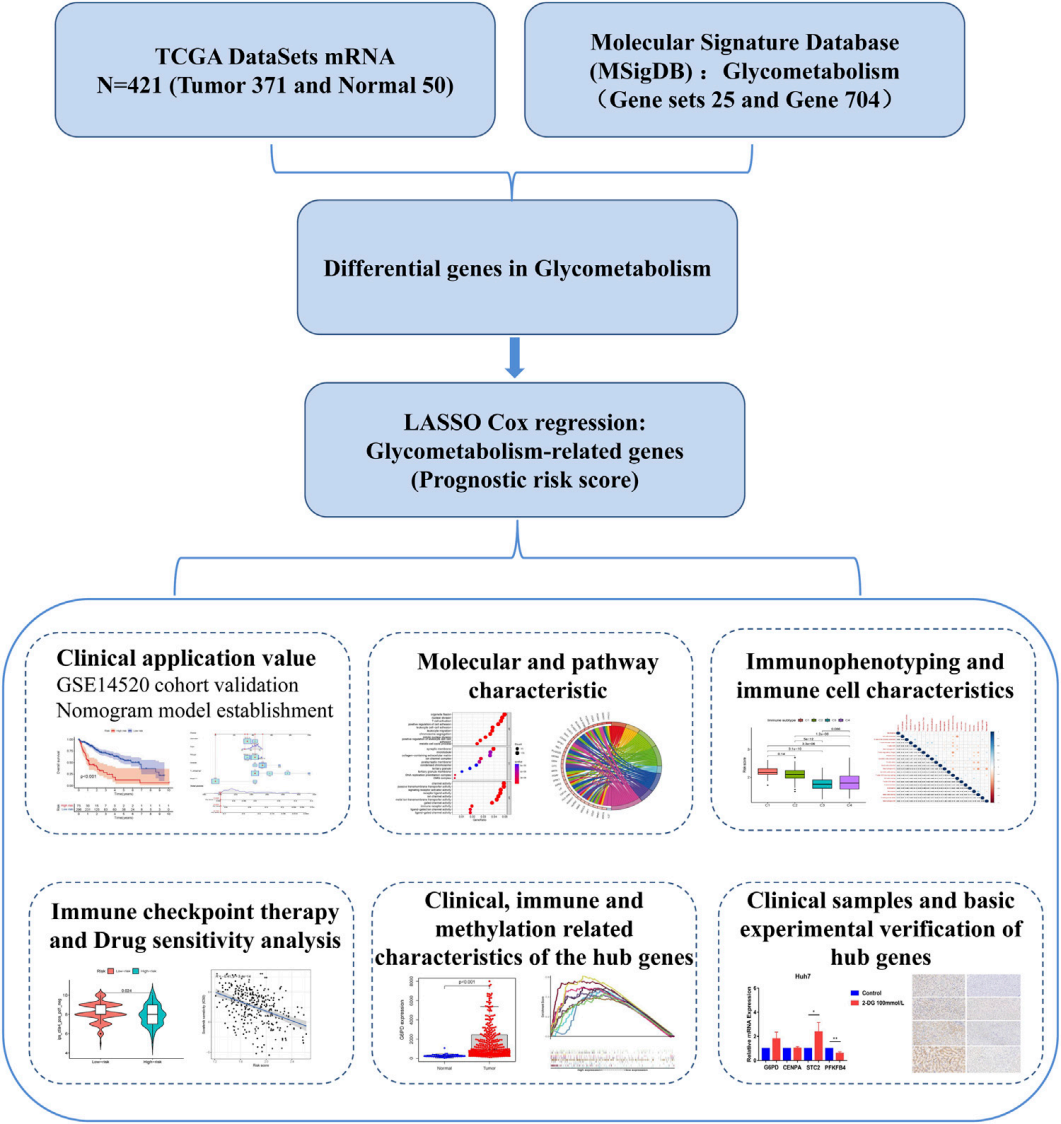
Construction and characterization of glycometabolism-related risk scores.

This study comprehensively assessed the glycometabolism patterns of patients with HCC and constructed a prognostic risk-score model for glycometabolism. We focused on all glycometabolism-related genes in HCC and constructed a prognostic risk score for these genes. We then characterized the molecular pathways and immune-related features of the prognostic risk score for glycometabolism-related genes, differentiated sorafenib-and 5-fluorouracil-resistant patients, and examined their responsiveness to immunosuppressant therapy. A technical roadmap of this study is shown in Figure 1. This study provides a new perspective for exploring the glycometabolism-immunity mechanism.

## Materials and methods

### Patients and datasets

A total of 421 liver hepatocellular carcinoma (LIHC) samples were downloaded from The Cancer Genome Atlas (TCGA) database, including 371 tumor samples and 50 normal samples with clinical and pathological staging data (https://portal.gdc.cancer.gov/). Among these, 369 tumor samples with complete clinical survival information were used as the training set for the model.

Microarray data for 242 HCC samples and their clinical information (GSE14520) were obtained from the Gene Expression Omnibus (GEO) database (https://www.ncbi.nlm.nih.gov/geo/), and the platform was GPL3921. The gene ID of each sample was converted into the corresponding gene symbol using an annotation platform. The average value was calculated if the same gene ID was mapped by multiple probes. Ultimately, 221 HCC samples with complete clinical survival information were included in the validation set. The clinical characteristics of the two cohorts are presented in Supplementary Table S1.

### Acquisition of glycometabolism genes

From the Molecular Signature Database (MSigDB; v7.5.1) (Subramanian et al., 2005), 25 glycometabolism-related gene sets were collected. After removing overlapping genes, 704 glycometabolism-related genes were obtained (Supplementary Table S2).

### Identification of glycometabolism-related hub genes

The differentially expressed genes related to glycometabolism between normal and tumor tissue samples were analyzed using the R package “limma.” Differentially expressed genes related to glycometabolism were obtained and analyzed using Metascape (http://metascape.org). Differentially expressed genes with |log2FC| > 2 and false discovery rate (FDR)< 0.05 were considered statistically significant.

Using the R package “glmnet,” we sequentially performed univariate Cox and least absolute shrinkage and selection operator (LASSO) Cox regression analyses and identified four glycometabolism-related genes associated with HCC survival.

### Construction and validation of the prognostic risk score

The prognostic risk score was obtained by multiplying the expression values of four glycometabolism-related hub genes by their weights in the LASSO Cox model and then adding them to calculate the risk score of each sample. The Kaplan-Meier (K-M) survival curve was used to evaluate the prognostic ability of the risk score in the TCGA and GEO databases. Univariate and multivariate Cox regression analyses were performed to verify the independent prognostic value of the risk score.

To reflect the clinical application and predictive value of the risk score, a nomogram model was constructed with risk scores and clinical indicators to predict patient prognosis at different times. The model’s calibration, accuracy, and clinical value were further evaluated using calibration, receiver operating characteristic (ROC), and decision curve analysis (DCA) curves.

### Comprehensive analysis of molecular and immune characteristics in different prognostic risk score subgroups

The R package “clusterProfiler” was used to analyze the enrichment of Gene Ontology (GO) and Kyoto Encyclopedia of Genes and Genomes (KEGG) pathways in different prognostic risk scores to determine the main biological characteristics and the enrichment of cellular functional pathways. A *p* value (*q* value) < 0.05 was defined as a statistically significant difference. Gene set enrichment analysis (GSEA) was also used to compare the differences in biological processes between the low-risk and high-risk groups, with the “hallmark all. v7.5. symbols” gene set as the internal parameter gene set. Statistical significance was set at *p* < 0.05 and FDR <0.05.

According to immune cell composition, 369 patients with HCC in the training set were divided into four immune subsets (Zhang et al., 2020a). To compare the immune characteristics of the 369 HCC samples with different prognostic risk scores, x-Cell (https://xcell.ucsf.edu/) and CIBERSORT (https://cibersort.stanford.edu/) were used to evaluate the relative proportions of 22 immune cells and the correlation between immune cells. Subsequently, the immune functions of the different prognostic risk scores were also compared. GSEA was also used to assess changes in immune-related pathways (“c7. immunesigdb. v7.3. symbols”) or biological processes with different prognostic risk scores.

To explore the prognostic value of different prognostic risk scores in patients after immunotherapy, the responsiveness to immune checkpoint PD-1 and CTLA-4 treatment was assessed using TCIA (https://tcia.at/home). The R package “pRRophetic” was used to analyze the drug sensitivity of patients with different prognostic risk scores.

### Quantitative real-time PCR

RNA was extracted from HepG2 and Huh7 cell lines using the RNeasy Plus Mini Kit (QIAGEN, Germany), and the RNA was reverse transcribed into cDNA using cDNA.

Synthesis kit (Thermo Fisher Scientific, United States), and then qRT-PCR was performed using Master Mix (SYBR Green; Lithuania). β-Actin was used as an internal control, and 2^−∆∆CT^ methodology was expressed as the relative expression of mRNA. Do at least three independent experiments. All mRNAs were purchased from the Synbio Technologies and the primers were listed in Supplementary Table S3.

### Immunohistochemistry

For immunohistochemical analysis, paraffin sections were performed on tumor tissues and adjacent tissues from patients with HCC. 4-µm-thick sections were soaked in xylene for 15 min, then deaffinity and rehydrated with an ethanol gradient, followed by incubation in 3% hydrogen peroxide for 15 min. Blocked with 10% goat serum for 30 min, incubated with primary antibodies to G6PD, CENPA, STC2, and PFKFB4 (abcam, 1:200) overnight at 4°C, and then used secondary antibodies conjugated to horseradish peroxidase (sigma, 1:200) and incubated for 30 min. Sections were scanned with panoramic scanning electron microscope, and positive staining was analyzed by ImageJ software (version 1.8.0).

### Statistical analysis

All statistical analyses were performed using the R software (version 4.1.3) and GraphPad Prism 7 software (version 7.0). Continuous variables were compared between the two groups using an independent *t*-test, and categorical data were compared using the χ^2^ test. Survival analysis was performed using the K-M survival analysis and log-rank test. Cox regression analysis was used to identify independent prognostic indicators predicting overall survival (OS) in HCC. Statistical significance was set at *p* < 0.05.

## Results

### Acquired glycometabolism-related hub genes in hepatocellular carcinoma patients

**FIGURE 2:**
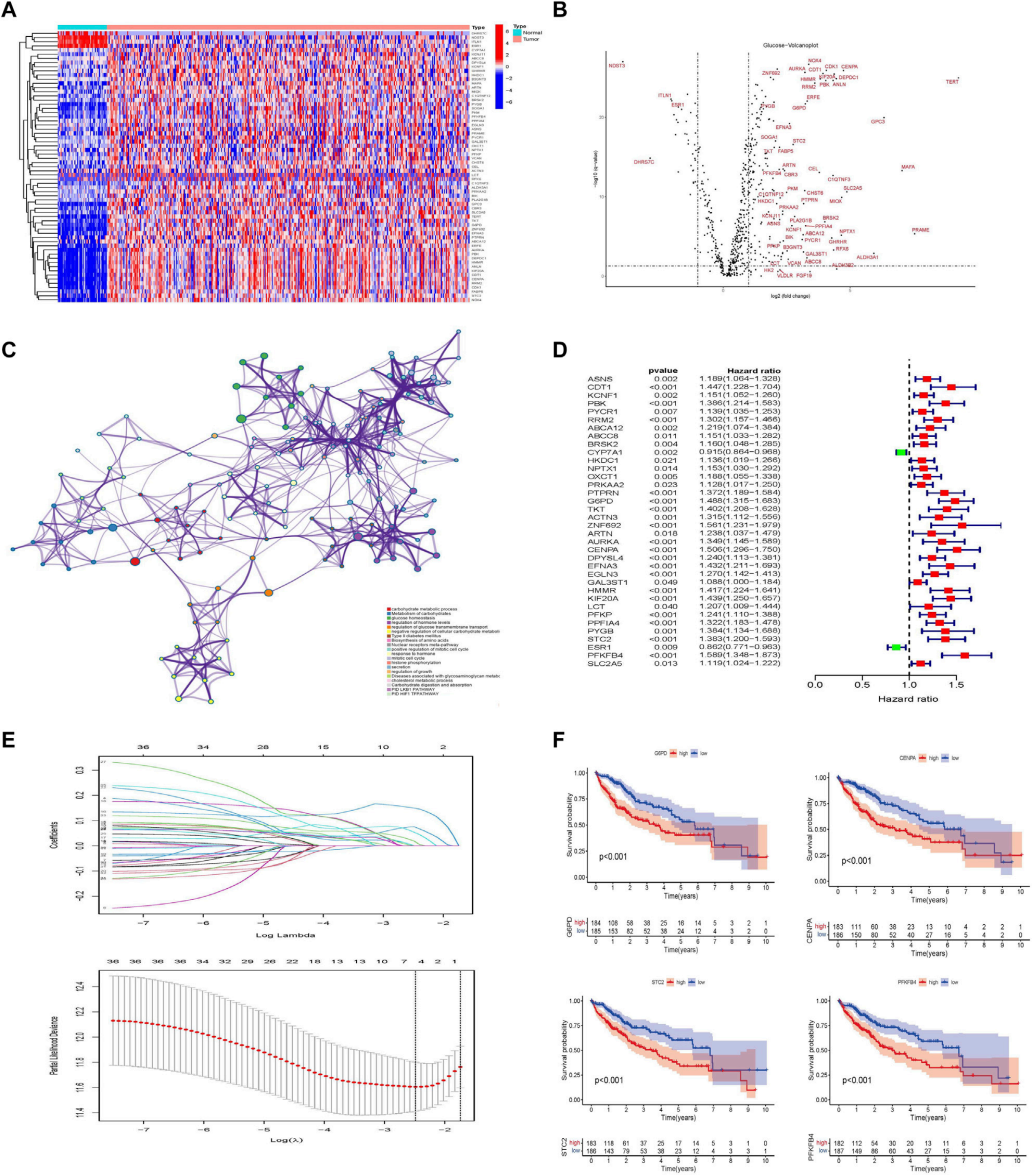
Establishment of glycometabolism-related hub genes in hepatocellular carcinoma. **(A)** Heatmap analysis of differentially expressed genes related to glycometabolism in the TCGA-LIHC cohort. **(B)** Volcano plot of the differentially expressed genes related to glycometabolism between tumor and normal tissues of HCC (|log2fc| > 2 and FDR <0.05). **(C)** Metascape analysis of pathway enrichment of differentially expressed genes related to glycometabolism. **(D)** Univariate Cox analysis of 36 differentially expressed genes related to glycometabolism. **(E)** LASSO regression to construct the hub gene of the prognostic risk scoring model. **(F)** Kaplan–Meier curve analysis of the effect of four hub genes on the overall survival of patients with HCC.

In the differential expression analysis (371 tumors vs. 50 normal samples), all genes in the TCGA database intersected with the glycometabolism-related genes obtained from the MSigDB gene set, and 678 glycometabolism-related genes were obtained. Subsequently, 65 differentially expressed genes (|log2FC| > 2 and FDR < 0.05) were identified between the normal and tumor groups (Figure 2A). The upregulated and downregulated differential genes with |log2FC| > 2 were displayed by volcano plot (Figure 2B). Furthermore, metascape functional enrichment analysis showed that 65 differentially expressed genes were significantly associated with “carbohydrate metabolic process,” “glucose homeostasis,” and “HIF1 TFPATHWAY” pathways (Figure 2C).

In the training set of the TCGA database, 36 differentially expressed genes related to glycometabolism were significantly associated with the prognosis of patients with HCC as revealed by Cox univariate analysis (*p* < 0.05; Figure 2D). LASSO regression was used to select lambda min as the model with the highest accuracy (Figure 2E). Four differentially expressed genes related to glycometabolism were more closely related to the OS of patients with HCC: glucose-6-phosphate dehydrogenase (G6PD), centromere protein A (CENPA), stanniocalcin 2 (STC2), and 6-phosphofructo-2-kinase/fructose-2,6-bisphosphatase 4 (PFKFB4). The median values of the four hub genes were divided into high and low expression groups. K-M survival analysis revealed that the OS rate of patients with HCC in the high expression group of hub genes was lower than that in the low expression group (*p* < 0.001; Figure 2F).

### Establishment and validation of the prognostic risk score and nomogram model

Subsequently, a prognostic risk score for all tumor samples was calculated using the formula: prognostic risk score = expression level of G6PD*(0.15) + expression level of CENPA*(0.075) + expression level of STC2*(0.018) + expression level of PFKFB4*(0.053). From the prognostic risk score, the cut-off value was calculated to be 2.12, and this value was used to divide the risk scores into high-and low-risk groups (Supplementary Figure S1).

**FIGURE 3:**
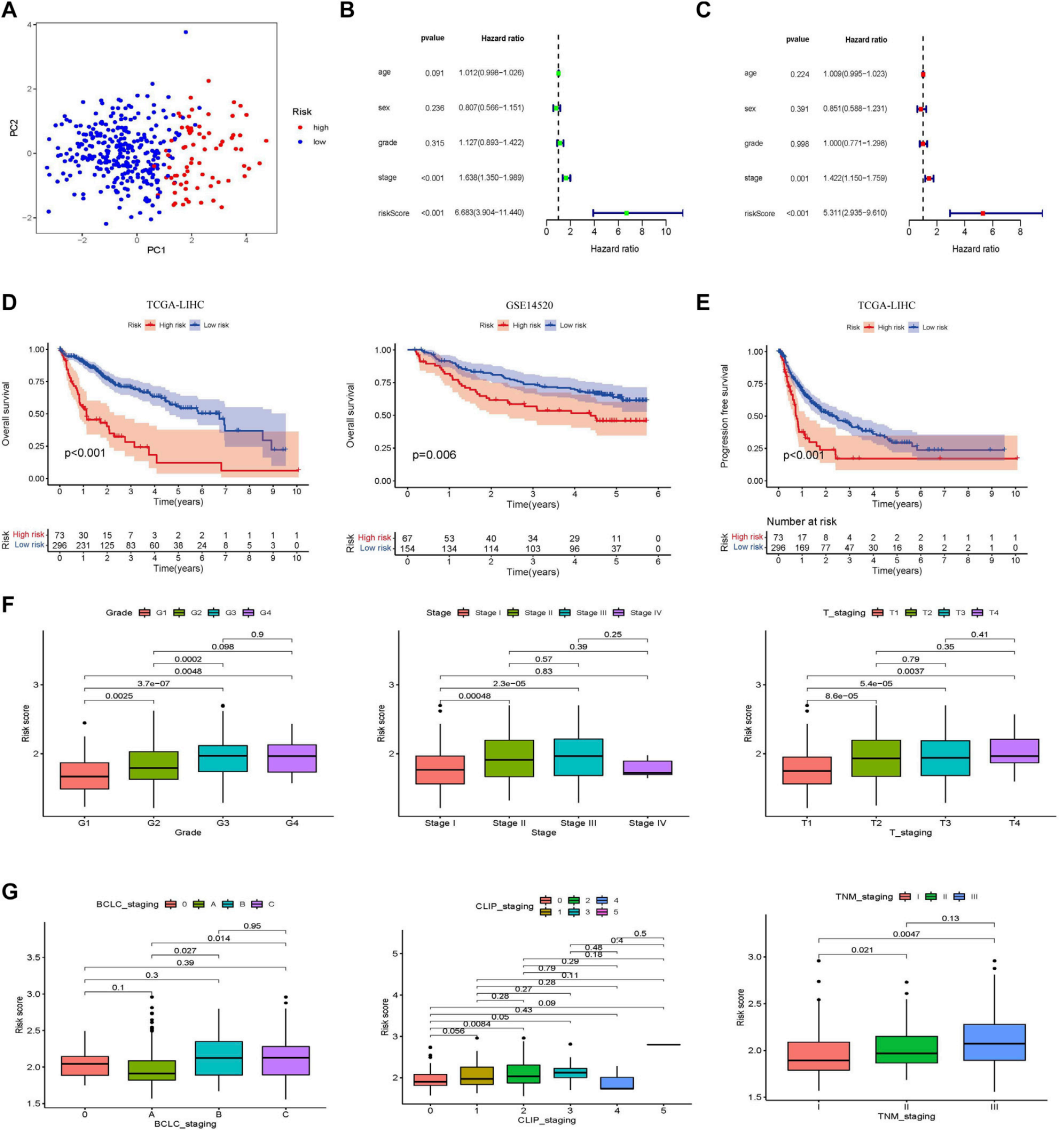
Establishment and validation of a prognostic risk score associated with glycometabolism. **(A)** Principal component analysis based on genes related to glycometabolism in patients with HCC. **(B,C)** Forest plot of univariate and multivariate Cox regression, respectively, in the TCGA-LIHC cohort. **(D)** Kaplan–Meier curve analysis comparing the overall survival between the low-risk and the high-risk groups of patients with HCC in the training and validation cohorts, respectively. **(E)** Kaplan-Meier curve analysis comparing the progression-free survival between the low-risk and high-risk groups of patients with HCC in the TCGA-LIHC cohort. **(F)** Comparison of the relationship between different glycometabolism-related risk scores and clinical stages in the TCGA cohort. **(G)** Comparison of the relationship between different glycometabolism-related risk scores and clinical stages in the GEO cohort.

Principal component analysis (PCA) was performed to identify significant differences between the low- and high-risk groups. The results showed that the prognostic risk score could distinguish between the high-and low-risk groups (Figure 3A). Clinically relevant indicators, including age, sex, grade, stage, and risk score, were included in the Cox univariate and multivariate analyses; only risk score and stage were independent predictors of OS in patients with HCC (*p* < 0.001; Figures 3B,C). Patients with different prognostic risk scores were divided into high- and low-risk groups according to the cutoff value (2.12). The OS of the patients in the high-risk group was lower than that of the patients in the low-risk group (*p* < 0.001; Figure 3D). The GSE14520 dataset (*n* = 221) was used to verify the effects of the different risk scores on patient survival. Patients in the high-risk group had lower survival rates than those in the low-risk group, which is consistent with the results from the TCGA dataset (*p* = 0.006). The effect of different risk scores on the progression-free survival (PFS) time of patients was also compared, and patients in the high-risk group were found to have shorter PFS times (*p* < 0.001; Figure 3E). Exploring the correlation between prognostic risk scores and clinical indicators furtherly, the training cohort revealed a gradual increase in the prognostic risk score with an increase in different grades, stages, and T (Primary Tumor) stages (Figure 3F). The results of the validation cohort were found to be similar to those of the validation cohort in other staging systems, such as Barcelona Clinic Liver Cancer (BCLC) staging, Cancer of the Liver Italian Program (CLIP) staging, and TNM (Tumor Node Metastasis) staging (Figure 3G). Subsequently, the area under the ROC curve of the prognostic risk score in the training cohort and the validation cohort was observed and compared. In the TCGA database training cohort, the area under curve (AUC) of the prognostic risk score at 1, 3, and 5 years was 0.790, 0.689, and 0.668; in the validation cohort, the AUC of the prognostic risk score at 1, 3, and 5 years was 0.722, 0.635, and 0.587 (Supplementary Figures S2A,B). We also compared the AUC values of the prognostic risk score with other biomarker signatures and found that the prognostic risk score was higher than the other signatures (Supplementary Figures S2C,D) (Zhang et al., 2020a; Zhang et al., 2020b; Pan et al., 2020; Dai et al., 2021). The results showed that compared with other signatures, the prognostic risk score had good predictive performance and the indicators were more concise.

A nomogram model integrating gender, age, stage, grade, T stage, and risk score was constructed and intuitively predicted the 1-, 3-, and 5-years survival rates of patients with HCC (Supplementary Figure S3A). Univariate and multivariate Cox regression analyses showed that the nomogram model was an independent prognostic factor for predicting the outcomes of patients with HCC (Supplementary Figures S3B,C). In addition, the 1-, 3-, and 5-years calibration curves showed that the nomogram model accurately predicted the prognosis of patients with HCC (Supplementary Figure S3D). The areas under the ROC curve were 0.789, 0.697, and 0.674 for 1, 3, and 5 years, respectively (Supplementary Figure S3E). The DCA curve showed that the nomogram model had a higher rate of clinical benefit than risk score and grade (Supplementary Figure S3F).

### Molecular and pathway characterization of different prognostic risk scores

**FIGURE 4:**
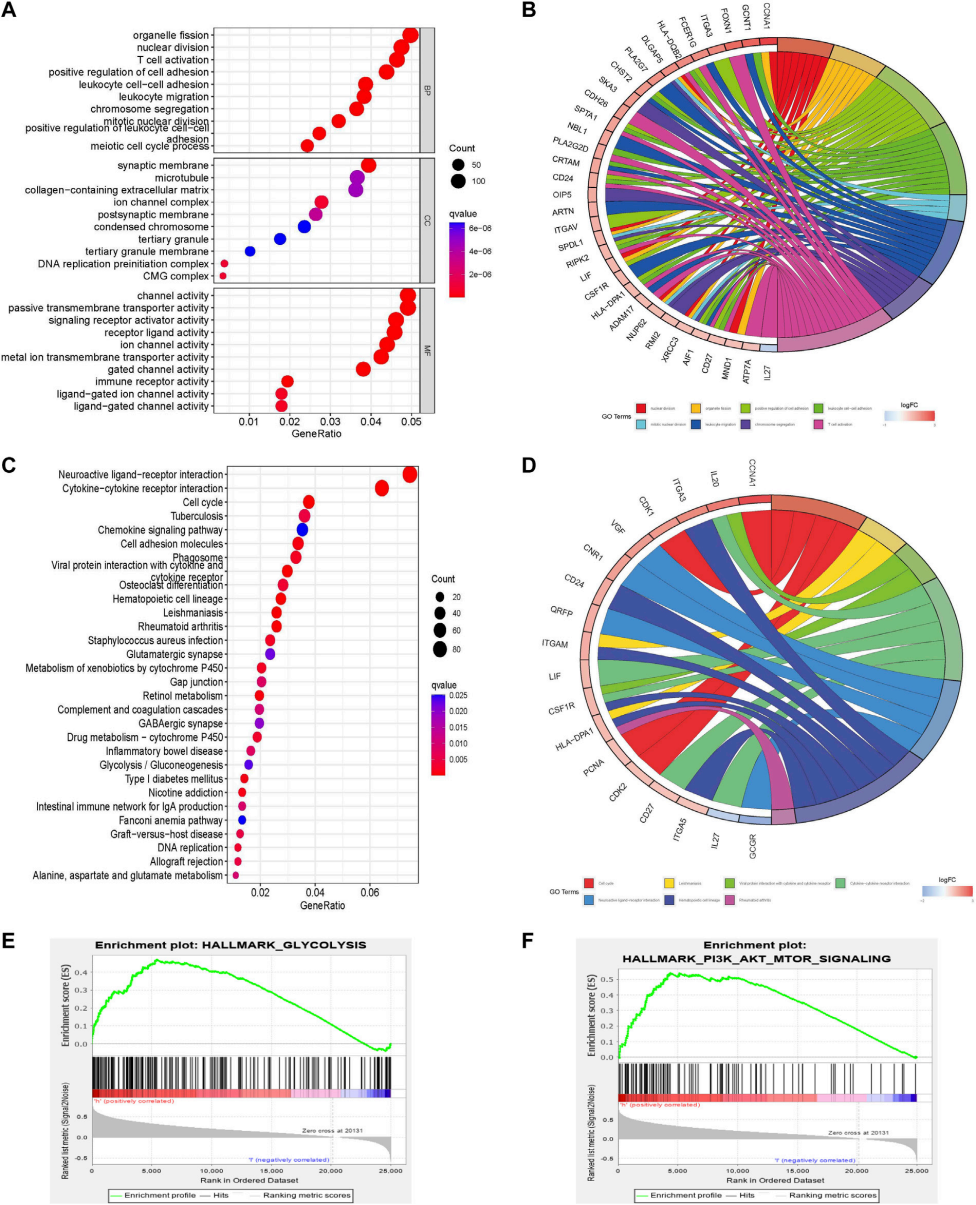
Pathway enrichment analysis comparing different risk scores associated with glycometabolism. **(A–D)** GO and KEGG enrichment analysis of the top 30 pathways and genes of different glycometabolism-related risk scores. **(E,F)** Comparison of the enrichment of the “hallmark all. v7.5. symbols” pathway in GSEA of different glycometabolism-related risk scores.

Molecular and pathway characteristics were further compared between the high- and low-risk groups. GO analysis revealed that nuclear division, mitotic nuclear division, and T cell activation pathways were significantly enriched in the high-risk group compared with the low-risk group. And related molecules enriched in high-risk groups include GCNT1, IL-27, and ATP7A, etc. (Figures 4A,B). Meanwhile, KEGG analysis showed that cell cycle, glycolysis and chemokine signaling pathways were significantly enriched in the high-risk group compared with the low-risk group and the related molecules included VGF, CDK2, and IL-27, etc. (Figures 4C,D). GSEA enrichment analysis also revealed that glycolysis (NES = 1.81, Nom *p* = 0.0, FDR *q* = 0.033) and PI3K AKT MTOR (NES = 1.73, Nom *p* = 0.002, FDR *q* = 0.046) pathways were mainly enriched in the high-risk group (Figures 4E,F; Supplementary Table S4).

### Immune cell characteristics and functions in different prognostic risk scores

**FIGURE 5:**
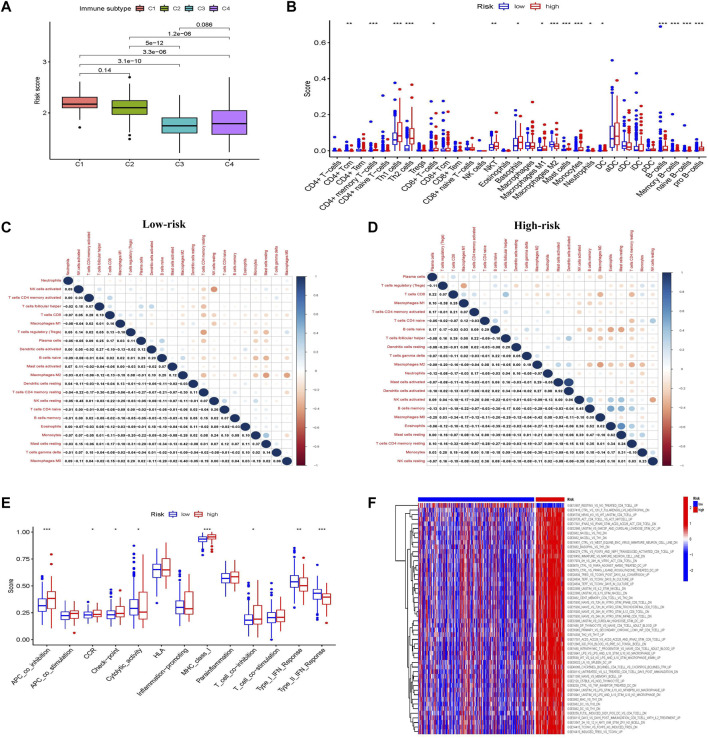
Characteristics, functions, and pathways of immune infiltrating cells in patients with liver cancer in different glycometabolism-related risk scores. **(A)** Analysis of the immune subtype of different risk scores. **(B)** The proportion of immune cells in the tumor microenvironment with different risk scores. **(C,D)** Correlations between immune-infiltrating cells in low-risk and high-risk scores, respectively. **(E)** Comparison of immune cell function between low-risk and high-risk scores. **(F)** Heatmap comparison of immune-related pathway enrichment between low-risk and high-risk scores. *p < 0.05, **p < 0.01, ***p < 0.001, ****p < 0.0001.

To observe the relationship between the prognostic risk score and immune subtype, HCC samples were divided into four immune subtypes: C1 (wound healing), C2 (IFN-γ dominant), C3 (inflammatory), and C4 (lymphocyte depleted). The C3 (inflammatory) immune subtype had the lowest risk score and best prognosis, consistent with the results of the original study (Figure 5A) (Vésteinn et al., 2018).

The composition and correlation of immune cells in different risk scores were analyzed, and it was found that CD4^+^ memory T-cells, Th1 cells, Th2 cells, basophils, and B cells were more abundant in the high-risk group, whereas macrophages were more abundant in the low-risk group (Figure 5B). Furthermore, the infiltrating immune cells in the low-risk group were mainly correlated with CD4^+^ memory T-cells and NK cells, while those in the high-risk group were mainly correlated with macrophages, B cells, and mast cells (Figures 5C,D).

The immune functions between the different risk scores were further explored. More immunosuppressive functions were observed in the high-risk group, such as APC coinhibition, checkpoint, MHC class I, and T-cell coinhibition (Figure 5E). Subsequently, the “c7.immunesigdb.v7.3. symbols” gene set downloaded from MSigDB was used to enrich gene set variation analysis and compare immune-related biological functions between the two groups. Interestingly, most immune-related genes were enriched in the high-risk group (Figure 5F).

### Immune checkpoint inhibitor treatment benefit and drug sensitivity in different prognostic risk scores

**FIGURE 6:**
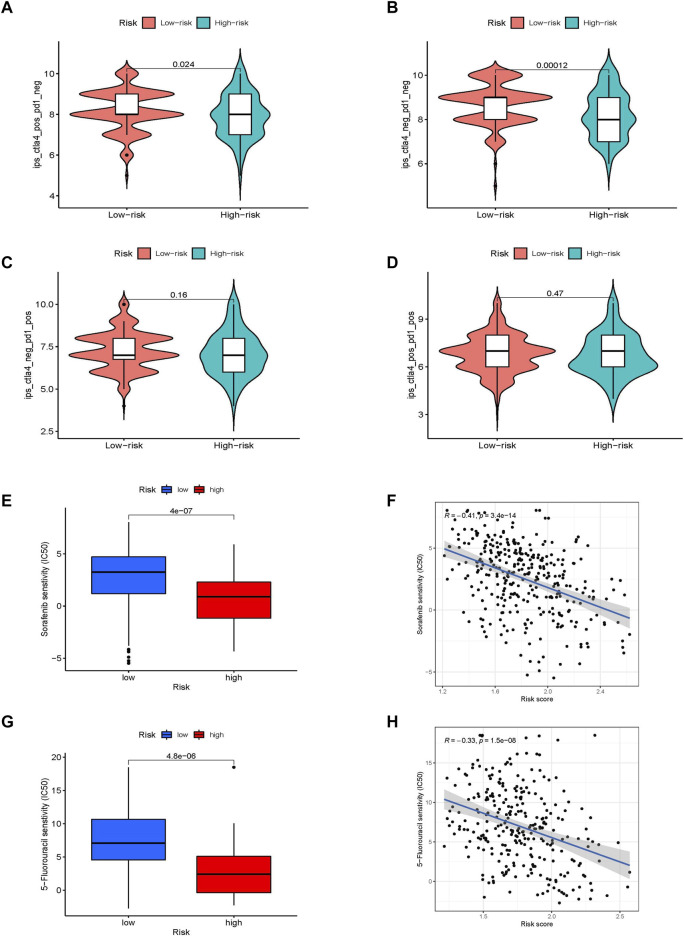
The value of different glycometabolism-related risk scores for ICI treatment and drug sensitivity. **(A–D)** Comparison of the responsiveness to anti-PD-1 and anti-CTLA-4 immunotherapy between samples in high-risk and low-risk groups. **(E–H)** Comparison of the sensitivities and associations to sorafenib and 5-fluorouracil drugs between high-risk and low-risk groups.

Major breakthroughs have been made in immune checkpoint therapies, particularly in PD-1 and CTLA-4 treatment. We investigated the ability of the prognostic risk score to predict response to immune checkpoint therapy. The results showed that although there was no difference in the clinical response to anti-PD-1 immunotherapy between the two groups, the response to anti-CTLA-4 immunotherapy in the low-risk group was higher than that in the high-risk group. Patients in the low-risk group were considered to be more suitable for CTLA-4 immune checkpoint therapy (Figures 6A–D). Because of the correlation between drug sensitivity and poor prognosis, we focused on the relationship between risk score and drug sensitivity. Sorafenib and 5-fluorouracil, the most widely used targeted drugs for HCC treatment, were selected to compare the drug sensitivity of the different risk groups. Interestingly, the high-risk group was found to be more sensitive to the targeted drugs sorafenib and 5-fluorouracil (*p* < 0.001), which were significantly negatively correlated with the risk score (*R* = −0.41, *p* < 0.001 and *R* = −0.33; *p* < 0.001, respectively; Figures 6E–H). This indicates that the glycometabolism-related risk score is a novel biomarker for assessing immunotherapy responsiveness and sensitivity to targeted drugs.

### Molecular pathways and immune characteristics of glycometabolism-related hub genes

**FIGURE 7:**
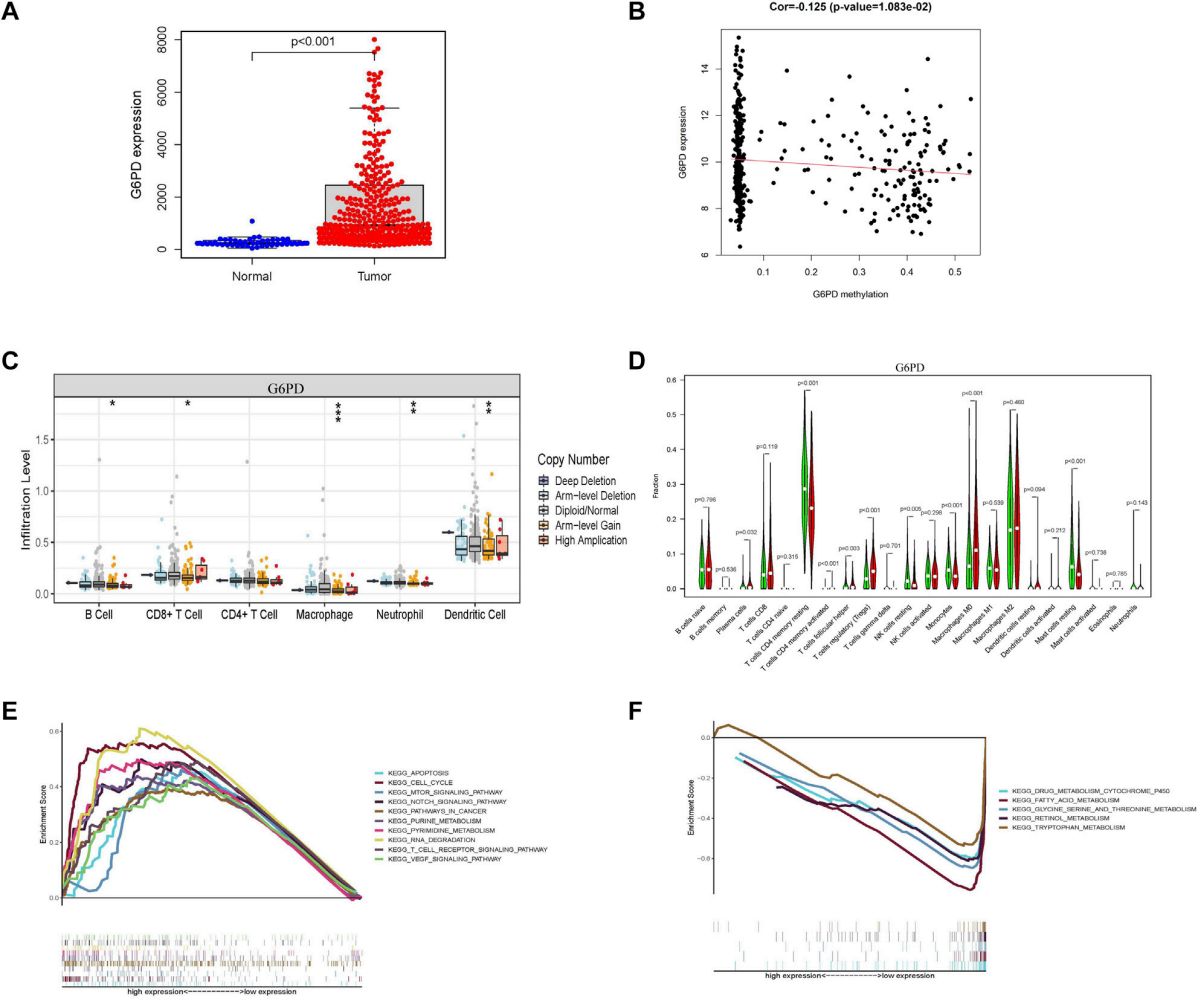
Characterization of G6PD as a hub gene related to glycometabolism-related risk score. **(A)** G6PD mRNA expression in tumor and normal tissues. **(B)** Correlation between G6PD expression level and its methylation level. **(C)** Differences in copy number variation of G6PD in different immune cells. **(D)** Comparison of immune infiltrating cells between high and low G6PD expression groups. **(E,F)** Multi-GSEA of KEGG pathway enrichment in high and low G6PD expression groups.

The hub genes related to glycometabolism in the risk score are also important. The levels of glycometabolism-related hub genes (G6PD, CENPA, STC2, and PFKFB4) in tumor samples were higher than those in normal samples (*p* < 0.001; Figure 7A; Supplementary Figure S4A). The relationships of the four glycometabolism-related hub genes with gender, clinical grade, stage, and T stage were investigated. The expression levels of the four glycometabolism-related hub genes significantly increased with an increase in tumor grade, stage, and T stage (Supplementary Figures S4B–E). Subsequently, we also focused on the methylation expression level of hub genes and found that the expression levels of G6PD (*p* = 1.083^−02^), CENPA (*p* = 7.08^−04^), STC2 (*p* = 3.507^−02^) and PFKFB4 (*p* = 3.541^−03^) genes were negatively correlated with their methylation levels (Figure 7B; Supplementary Figures S5A–C). The G6PD gene has copy number variations in B cells, CD8^+^ T-cells, macrophages, neutrophils, and dendritic cells (Figure 7C, Supplementary Figures S5D–F). At the same time, the high G6PD expression group was mainly composed of regulatory T-cells and macrophage M0 cells, which was significantly different from the composition in the low expression group (*p* < 0.001; Figure 7D; Supplementary Figures S5G–I). Multiple GSEA analysis of G6PD showed that mTOR signaling, Notch signaling, and cancer pathways were mainly enriched in the high expression group, whereas other metabolic pathways, such as drug metabolism, fatty acid metabolism, and tryptophan metabolism were enriched in the low expression group (Figures 7E,F).

### Clinical samples and basic experimental verification of hub genes

**FIGURE 8:**
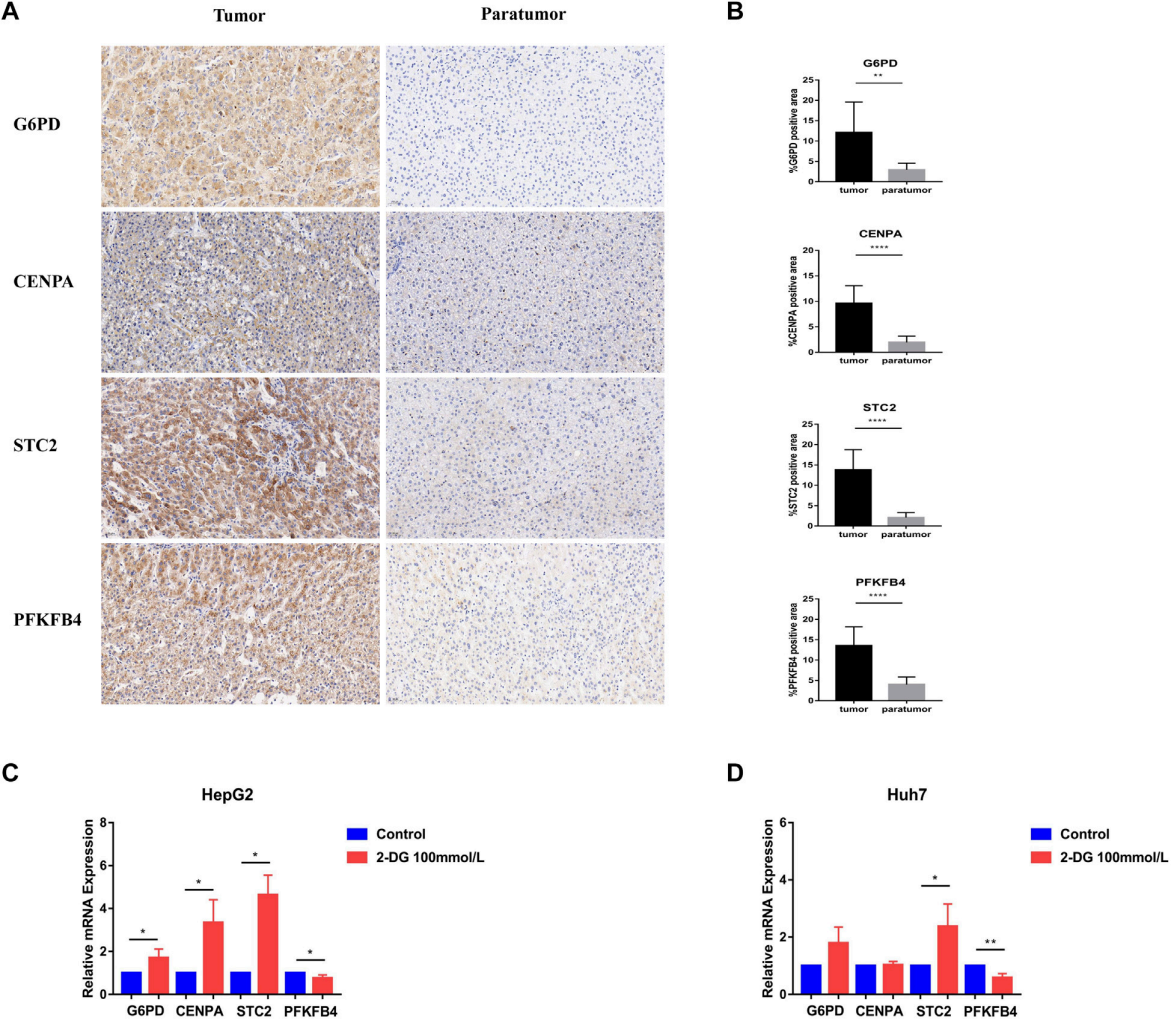
Experimental verification of glycometabolism-related genes. **(A,B)** Immunohistochemistry showed the G6PD, CENPA, STC2, and PFKFB4 genes expression and positive rate in tumor tissues (*n* = 13) and adjacent tissues (*n* = 13). **(C,D)** In HepG2 and Huh7 cell lines, G6PD, CENPA, STC2, and PFKFB4 mRNA expression levels were observed in the glycolysis inhibitor treated group (2-DG) and the control group.*p < 0.05, **p < 0.01, ***p < 0.001, ****p < 0.0001.

Further, we verified the results in the database through clinical samples and cell line experiments. We selected patients who had undergone hepatectomy for HCC to take tumor tissues and adjacent tissues for immunohistochemical analysis. It was found that the details of four glycometabolism-related genes in tumor tissues were significantly higher than those in adjacent tissues (*p* < 0.01; Figures 8A,B). Subsequently, the expression of four glycometabolism-related genes was detected in HepG2 and Huh7 cell lines after incubation for 48 h and blocking with glycolysis blocker 2-DG. After blocking the glycolytic pathway, only the expression of PFKFB4 decreased in both HepG2 and Huh7 cell lines (*p* < 0.05; Figures 8C,D), while STC2 increased in both HepG2 and Huh7 cell lines (*p* < 0.05). The expression of G6PD and CENPA increased only in HepG2 cell line (*p* < 0.05).

## Discussion

Glucose metabolism is important for all cells to maintain their activity (Bose and Le, 2018). Reprogramming of glucose metabolism can lead to tumor cell progression, metastasis, and recurrence (Peng et al., 2021). However, changes in glucose metabolism in immune cells have attracted increasing attention in immune metabolism. Targeted glucose metabolism may become a promising method for regulating immunity and improving the efficacy of immunotherapy, and may play an important role in the field of tumors and viruses (Hay, 2016; O’Neill et al., 2016). Increasing evidence has revealed the relationship between glucose metabolism reprogramming of immune effector cells, such as T-cells and NK cells, and tumor occurrence and development (Cong et al., 2018; Kang and Tang, 2020). In the past, glycometabolism-related genes have been used to evaluate the prognosis of patients with prostate cancer, gastric cancer, ovarian cancer, and lung adenocarcinoma (Huang et al., 2019; Liu et al., 2020; Zhang et al., 2021a; He et al., 2021). However, there is no prognostic model based on glycometabolism-related genes that evaluates the survival and immune-related characteristics of patients with HCC. Although there are some studies on metabolism-associated molecular classification in HCC (Yang et al., 2020), it is necessary to further focus on the target of glycometabolism, which is beneficial for improving clinical efficacy.

With the rapid development of transcriptomics and bioinformatics, an increasing number of gene markers have become available. The prognostic risk scoring system and nomogram model of glycometabolism-related genes in HCC tumor and normal samples from TCGA and GEO cohorts were established through LASSO Cox regression analysis. The prognostic risk score of glycometabolism-related genes can clarify the roles of hub genes in HCC. The nomogram model intuitively predicted the survival rates of patients at different follow-up times (Balachandran et al., 2015). The OS rate of the patients with HCC in the high-risk group was lower than low-risk group. Both the training and validation groups showed consistent results. The prognostic risk score of glycometabolism-related genes can effectively distinguish high-risk groups and has a particular guiding role in the survival and prognosis of patients with HCC.

The glycometabolism-related prognostic risk scores included G6PD, CENPA, STC2, and PFKFB4. G6PD is the core enzyme of the pentose phosphate pathway (PPP) in glucose metabolism and is involved in tumor cell growth, invasion, and metastasis (Yang et al., 2021). Previous studies have reported that high Nrf2 expression can enhance the expression of G6PD and HIF-1 in breast cancer cells. Nrf-2 activates antioxidant enzymes and upregulates Notch-1 through the G6PD/HIF-1 pathway, thereby affecting the proliferation of breast cancer cells (Zhang et al., 2019a). At the same time, G6PD overexpression was found to be associated with an increase in mTORC1 activity in blood tumors such as acute myeloid leukemia, and also predicts a poor prognosis (Poulain et al., 2017). In this study, we found that the expression of G6PD in tumor tissues was significantly higher than that in normal tissues, which was also confirmed by immunohistochemical analysis. Meanwhile, it was found that the Notch and mTOR signaling pathways were significantly enriched in the high G6PD expression group, which was consistent with previous reports. CENPA was found to be highly expressed in a variety of tumors and is associated with poor prognosis (Saha et al., 2020; Han et al., 2021). CENPA acts as an upstream transcriptional activator of the karyopherin α2 subunit gene (KPNA2), indirectly promoting tumor cell growth and glycolysis in patients with colon cancer (Liang et al., 2021). *In vitro* studies have also shown that CENPA can activate the Wnt/β-catenin signaling pathway and promote the proliferation and metastasis of renal cell carcinoma (Wang et al., 2021a). Glycolysis was the main factor that drives tumor development. STC2 participates in glycolysis-related pathways and phosphorus metabolism (Li et al., 2021). It was highly expressed in pancreatic, lung, colon, and breast cancer and other malignant tumors and is associated with poor prognosis (Na et al., 2015; Zhang et al., 2019b; Jiang et al., 2019; Lin et al., 2019). However, PFKFB4 was a key regulatory enzyme of glycolytic synthesis. In breast, bladder, and pancreatic cancer and other malignant tumors, targeting the glycolysis pathway mediated by PFKFB4 can inhibit the growth and invasion of tumor cells (Zhang et al., 2016; Dasgupta et al., 2018; Zhang et al., 2021b). Our basic experiments found that glycolysis inhibitors significantly inhibited the level of PFKFB4 mRNA, while other hub genes are not directly controlled by glycolysis pathway, and there may be other metabolic pathways. It was found in the tumor tissue of HCC patients that compared with the adjacent tissue, the tumor tissue significantly overexpressed 4 glycometabolism-related genes. Therefore, glycometabolism-related risk score was a biomarker of tumor promotion and is significantly correlated with poor prognosis.

Understanding the immune cell composition, molecular pathways, and immune functions of different glycometabolism-related risk scores in TME can improve immunotherapy. The relative proportions of 31 immune cells were further assessed using x-Cell, and the correlation between infiltrating immune cells was assessed using CIBERSORT, which were compared in different glycometabolism-related risk scores. The results showed that the main enriched immune cells in the high-risk group were inflammatory cells such as Th1, Th2, basophils, M2 macrophages, and B cells. Through GO and KEGG pathway enrichment analysis, the IL-27 pathway was found to be enriched in the high-risk group. It has been reported that IL-27 can affect multiple effector cells in innate and adaptive immunity (Villarino et al., 2005; Li et al., 2020; Dong et al., 2021). Therefore, enrichment of the IL-27 pathway may be associated with higher scores of CD8^+^T and NKT cells in the high-risk group. It was suggested that glycometabolism-related risk scores and immune inflammatory function may be mediated by IL-27 signaling. The traditional understanding of IL-27 is that its main response cells are immune cells. A recent study in the journal Nature found that IL-27 promotes the browning of adipose tissue by up-regulating the expression of Uncoupling Protein 1 (UCP1) to promote heat production and energy consumption, thereby reducing obesity (Wang et al., 2021b). This was also the latest understanding of the corresponding non-immune cells of IL-27. The results of this study also showed that the high-risk group was mainly involved in immunosuppressive function, including APC coinhibition, checkpoint, and T-cell coinhibition. The data suggested that the high-risk group has characteristics of immune inflammatory cells and immunosuppressive function, and the immune cell infiltration and inflammatory characteristics lead to a poor prognosis in the high-risk group.

Since glycometabolism-related risk scores were associated with poor prognosis in HCC, we explored the relationship between risk scores and resistance to ICI (PD-1 and CTLA-4) and targeted drugs (sorafenib and 5-fluorouracil). PD-1 and CTLA-4 were gradually used for immune checkpoint inhibitors to treat liver cancer (Finkelmeier et al., 2018; Fessas et al., 2020). Sorafenib was a first-line targeted drug used for the treatment of liver cancer. Therefore, it was necessary to analyze their clinical reactivity and drug resistance to better guide clinical medication. We analyzed the relationship between different glycometabolism-related risk scores and immunophenoscore in patients with HCC. The low-risk group had a higher response to anti-CTLA-4 treatment, indicating that the response to ICI was better in the low-risk group. Subsequently, analysis of sorafenib and 5-Fluorouracil drug resistance revealed that the half maximal inhibitory concentration (IC_50_) of the high-risk group was lower than that of the low-risk group, indicating that drug resistance was less likely to occur in the high-risk group. Glycometabolism-related risk scores provided a possible basis for clinical treatment, and further experimental and clinical verification is needed.

The current study has certain limitations. First, the established prognostic risk score for glycometabolism needs to be validated in a larger multicenter cohort. Second, the downloading of relevant data from public databases is very limited, and it is unknown whether the patient has other metabolic problems. Third, it is necessary to further explore the mechanism of glycometabolism and immunity on the progression and prognosis of HCC. In the future research work, we will pay attention to the clinical application value of this prognostic risk score, and explore the application prospects of glucose metabolism in the field of tumor immunity, which is very instructive and valuable work.

In conclusion, we constructed a prognosis risk score based on glycometabolism-related genes that can predict OS and PFS in patients with HCC and reflect the responsiveness to immune infiltrating cells and immunotherapy in patients. In addition, the prognostic risk score may be a potential biomarker in the field of immunometabolism. Further clinical and experimental studies are required to confirm these findings.

## Data Availability

The datasets presented in this study can be found in online repositories. The names of the repository/repositories and accession number(s) can be found in the article/Supplementary Material.
