# Supplementary material for: Glycometabolism-related gene signature of hepatocellular carcinoma predicts prognosis and guides immunotherapy
Source: Front Cell Dev Biol. 2022 Jul 22;10:940551. doi: 10.3389/fcell.2022.940551 (PMC9354664; doi:10.3389/fcell.2022.940551)
Supplement: Supplementary file 1 [file DataSheet1.docx]

Table S1. Clinical characteristics of the TCGA and GEO cohorts.

|  |  | Training set |  | Validation set |
| --- | --- | --- | --- | --- |
| Variable |  | TCGA(n=369) |  | GSE14520(n=221) |
| Age(y) |  |  |  |  |
|  | >65 | 137 |  | 21 |
|  | ≤65 | 232 |  | 200 |
| Gender |  |  |  |  |
|  | Male | 248 |  | 191 |
|  | Female | 121 |  | 30 |
| Stage |  |  |  |  |
|  | I | 175 |  | NA |
|  | II | 89 |  | NA |
|  | III | 100 |  | NA |
|  | IV | 5 |  | NA |
| Grade |  |  |  |  |
|  | G1 | 55 |  | NA |
|  | G2 | 180 |  | NA |
|  | G3 | 122 |  | NA |
|  | G4 | 12 |  |  |
| T staging | |  |  |  |
|  | T1 | 180 |  | NA |
|  | T2 | 95 |  | NA |
|  | T3 | 80 |  | NA |
|  | T4 | 13 |  | NA |
|  | unknow | 1 |  | NA |
| TNM staging | |  |  |  |
|  | I | NA |  | 93 |
|  | II | NA |  | 77 |
|  | III | NA |  | 49 |
|  | unknow | NA |  | 2 |
| BCLC staging | |  |  |  |
|  | 0 | NA |  | 20 |
|  | A | NA |  | 148 |
|  | B | NA |  | 22 |
|  | C | NA |  | 29 |
|  | unknow | NA |  | 2 |
| CLIP staging | |  |  |  |
|  | 0 | NA |  | 91 |
|  | 1 | NA |  | 70 |
|  | 2 | NA |  | 33 |
|  | 3 | NA |  | 9 |
|  | 4 | NA |  | 3 |
|  | 5 | NA |  | 1 |
|  | unknow | NA |  | 14 |

Table S2. List of glycometabolism-related gene sets from the Molecular Signature Database.

|  | Standard name | Systematic name | genes |
| --- | --- | --- | --- |
| 1 | CUI_GLUCOSE_DEPRIVATION | M9780 | 61 |
| 2 | GOBP_CELLULAR_GLUCOSE_HOMEOSTASIS | M14255 | 155 |
| 3 | GOBP_GLUCOSE_6_PHOSPHATE_METABOLIC_PROCESS | M11917 | 24 |
| 4 | GOBP_GLUCOSE_IMPORT | M13142 | 79 |
| 5 | GOBP_GLYCOLYTIC_FERMENTATION | M29123 | 5 |
| 6 | GOBP_NEGATIVE_REGULATION_OF_GLUCOSE_TRANSMEMBRANE_TRANSPORT | M22686 | 22 |
| 7 | GOBP_POSITIVE_REGULATION_OF_GLUCOSE_IMPORT | M40449 | 37 |
| 8 | GOBP_POSITIVE_REGULATION_OF_GLUCOSE_METABOLIC_PROCESS | M16946 | 42 |
| 9 | GOBP_POSITIVE_REGULATION_OF_GLUCOSE_TRANSMEMBRANE_TRANSPORT | M22685 | 44 |
| 10 | GOBP_REGULATION_OF_GLUCOSE_IMPORT | M34192 | 60 |
| 11 | GOBP_REGULATION_OF_GLUCOSE_METABOLIC_PROCESS | M13722 | 123 |
| 12 | GOBP_REGULATION_OF_GLUCOSE_TRANSMEMBRANE_TRANSPORT | M22684 | 78 |
| 13 | GOBP_REGULATION_OF_TRANSCRIPTION_BY_GLUCOSE | M23770 | 7 |
| 14 | GOBP_UDP_GLUCOSE_METABOLIC_PROCESS | M29023 | 5 |
| 15 | HP_IMPAIRED_GLUCOSE_TOLERANCE | M38615 | 30 |
| 16 | KEGG_GLYCOLYSIS_GLUCONEOGENESIS | M11521 | 62 |
| 17 | MOOTHA_GLYCOLYSIS | M16111 | 21 |
| 18 | REACTOME_GLUCOSE_METABOLISM | M1870 | 92 |
| 19 | BIOCARTA_GLYCOLYSIS_PATHWAY | M15109 | 3 |
| 20 | HALLMARK_GLYCOLYSIS | M5937 | 200 |
| 21 | REACTOME_GLYCOLYSIS | M5113 | 72 |
| 22 | WP_AEROBIC_GLYCOLYSIS | M39816 | 12 |
| 23 | WP_GLYCOLYSIS_AND_GLUCONEOGENESIS | M39474 | 45 |
| 24 | WP_GLYCOLYSIS_IN_SENESCENCE | M40053 | 11 |
| 25 | WP_HIF1A_AND_PPARG_REGULATION_OF_GLYCOLYSIS | M39394 | 8 |

Table S3. Primers used for real time PCR.

| Gene | Sequences |
| --- | --- |
| G6PD-human-S | 5′-CTGTTCCGTGAGGACCAGATCT-3′ |
| G6PD-human-R | 5′-TGAAGGTGAGGATAACGCAGGC-3′ |
| CENPA-human- S | 5′-GGCGGAGACAAGGTTGGCTAAA-3′ |
| CENPA-human- R | 5′-GGCTTGCCAATTGAAGTCCACAC-3′ |
| STC2-human- S | 5′-GCATGACTTTTCTGCACAACGCT-3′ |
| STC2-human- R | 5′-GGCTTATGCAGCCGAACCTGTG -3′ |
| PFKFB4-human -S | 5′-GATCCTGAGGTCATAGCTGCCA-3′ |
| PFKFB4-human- R | 5′-CTATCCAGGTCCTCATCTAGCG-3′ |
| b-actin-human-S | 5′-GAGCTACGAGCTGCCTGACG-3′ |
| b-actin-human-R | 5′-GTAGTTTCGTGGATGCCACAG-3′ |

Table S4. GSEA of different glycometabolism-related risk scores in the "hallmark all. v7.5. symbols" gene set. *P* < 0.05 and FDR < 0.05 were statistically significant.

| NAME | ES | NES | NOM p-val | FDR q-val | FWER p-val |
| --- | --- | --- | --- | --- | --- |
| HALLMARK_E2F_TARGETS | 0.765042 | 1.993387 | 0 | 0.04162 | 0.019 |
| HALLMARK_MTORC1_SIGNALING | 0.598809 | 1.986759 | 0 | 0.02081 | 0.019 |
| HALLMARK_MYC_TARGETS_V1 | 0.701405 | 1.933335 | 0 | 0.022843 | 0.029 |
| HALLMARK_G2M_CHECKPOINT | 0.709295 | 1.891005 | 0 | 0.028525 | 0.049 |
| HALLMARK_MYC_TARGETS_V2 | 0.706291 | 1.87223 | 0.00201207 | 0.026587 | 0.055 |
| HALLMARK_UNFOLDED_PROTEIN_RESPONSE | 0.595598 | 1.817184 | 0 | 0.036436 | 0.085 |
| HALLMARK_GLYCOLYSIS | 0.470755 | 1.807198 | 0 | 0.033516 | 0.09 |
| HALLMARK_DNA_REPAIR | 0.587877 | 1.765875 | 0.00407332 | 0.041979 | 0.117 |
| HALLMARK_UV_RESPONSE_UP | 0.454591 | 1.745278 | 0 | 0.045706 | 0.137 |
| HALLMARK_PI3K_AKT_MTOR_SIGNALING | 0.541863 | 1.729744 | 0.00208768 | 0.046935 | 0.157 |


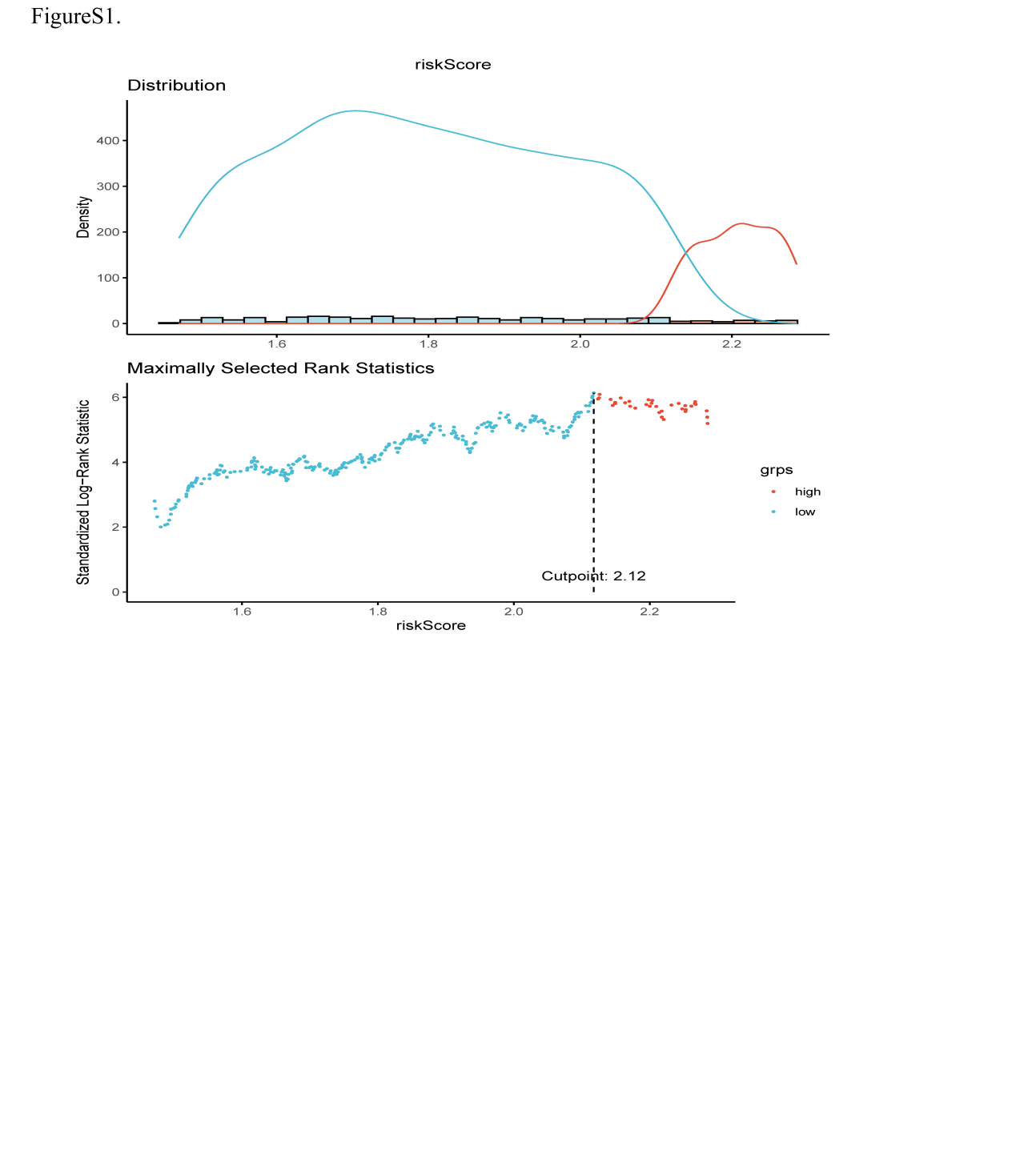


Figure S1. The cutoff value for glycometabolism-related prognostic risk score was calculated and divided into high-risk group and low-risk group.


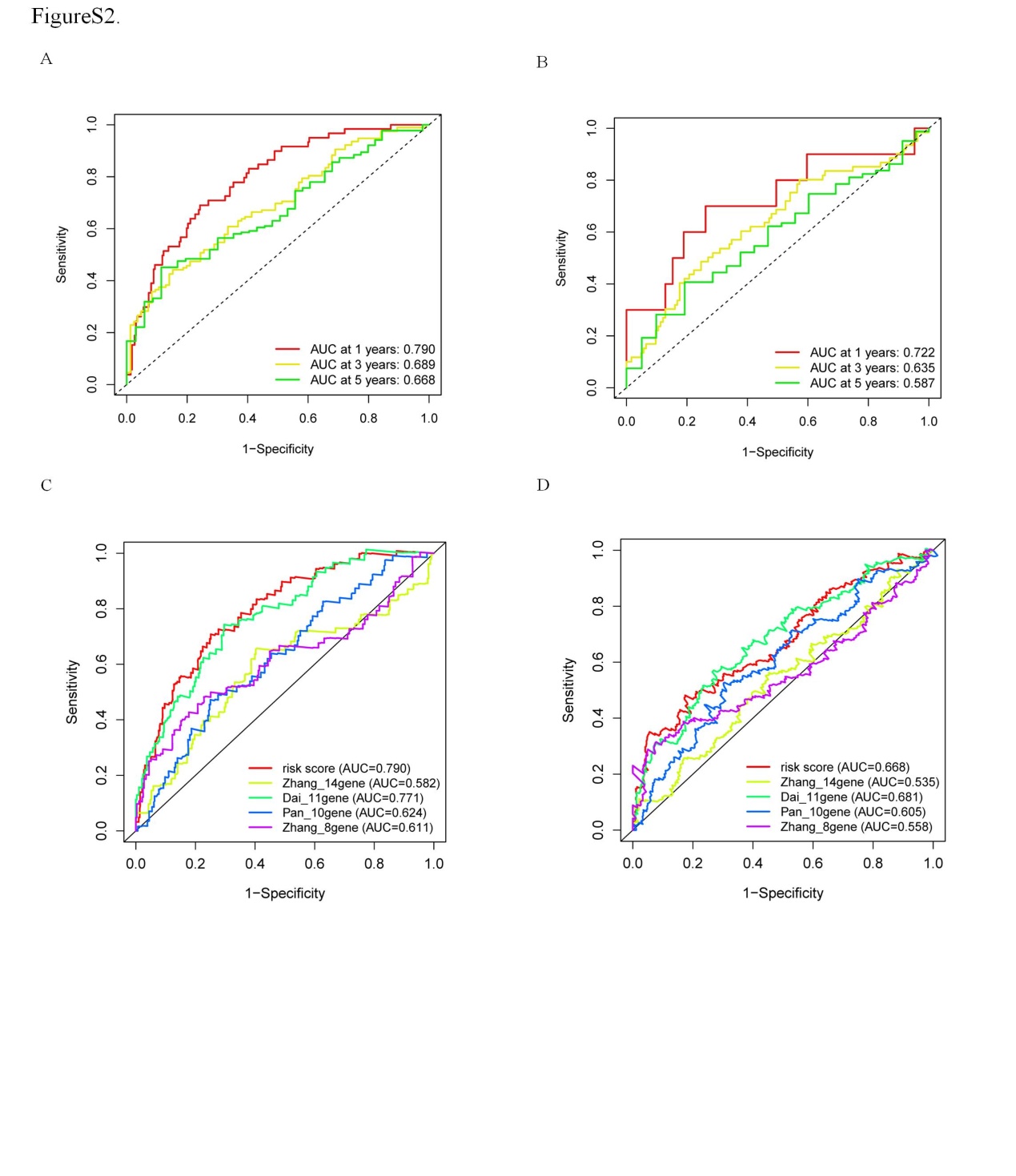


Figure S2. Prediction accuracy of prognostic risk score. (A-B) The area under the ROC curve at 1, 3 and 5 years in the TCGA training and GEO validation cohort. (C-D) The area under the ROC curve at 1 and 5 years for risk scores compared with other liver cancer related biomarkers.


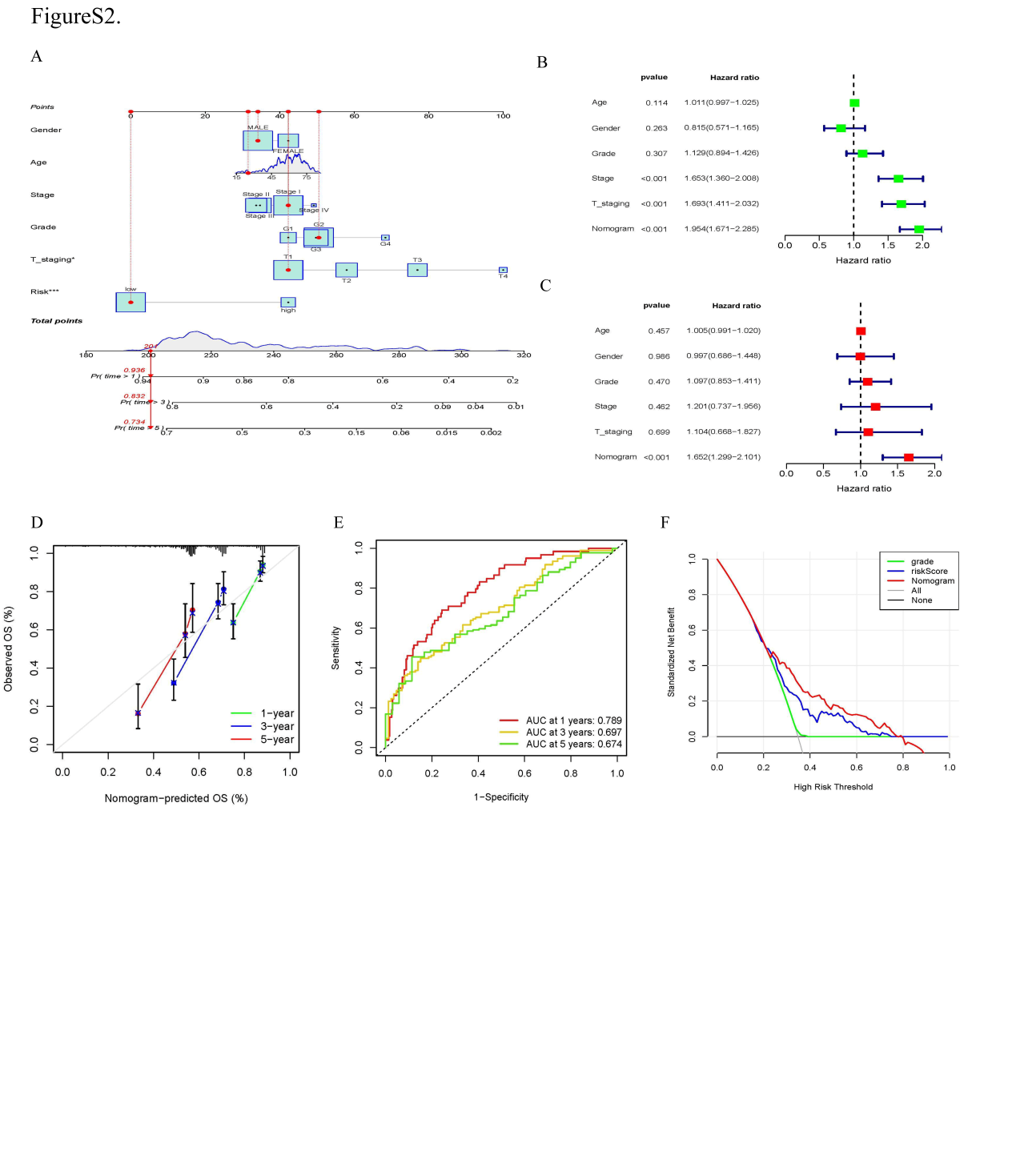


Figure S3. Establishment of a nomogram model and evaluation of its predictive performance. (A) Nomogram model combined with clinical indicators in predicitng the prognosis of patients with HCC in the TCGA cohort. (B-C) Forest plot showing univariate and multivariate Cox regression analysisof the nomogram model, respectively. (D) 1-, 3- and 5-year calibration curves of the nomogram model. (E) 1-, 3- and 5- ROC curves of the nomogram model. (F) The DCA curve shows nomogram, risk score and clinical grade.


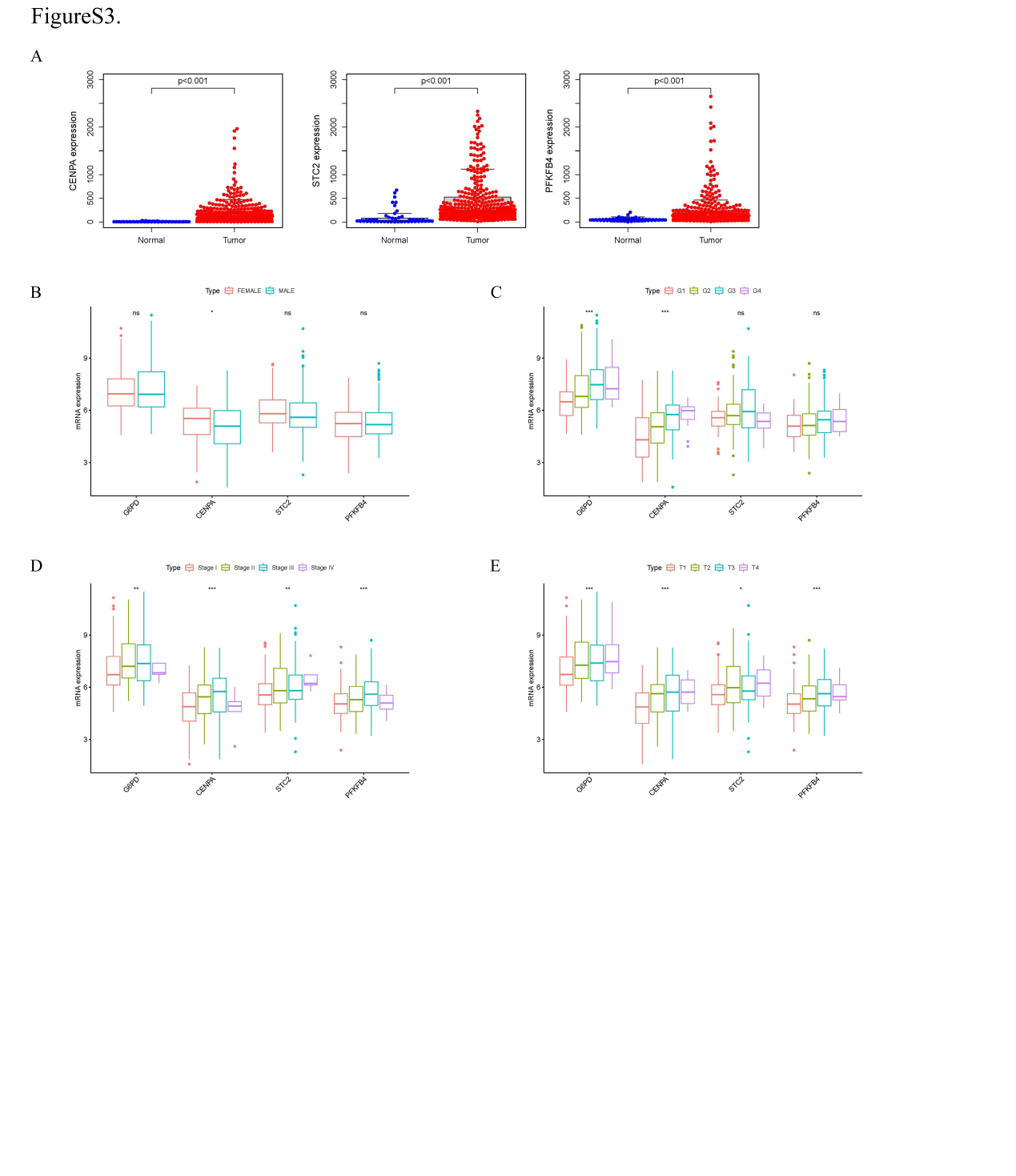


Figure S4. Clinical characteristics of hub genes in the glycometabolism-related risk score. (A) The expression level of CENPA, STC2, and PFKFB4 mRNA in tumor and normal tissues. (B-E) The expression levels of four hub genes (G6PD, CENPA, STC2, and PFKFB4 mRNA) were in gender, grade, stage and T stage respectively.


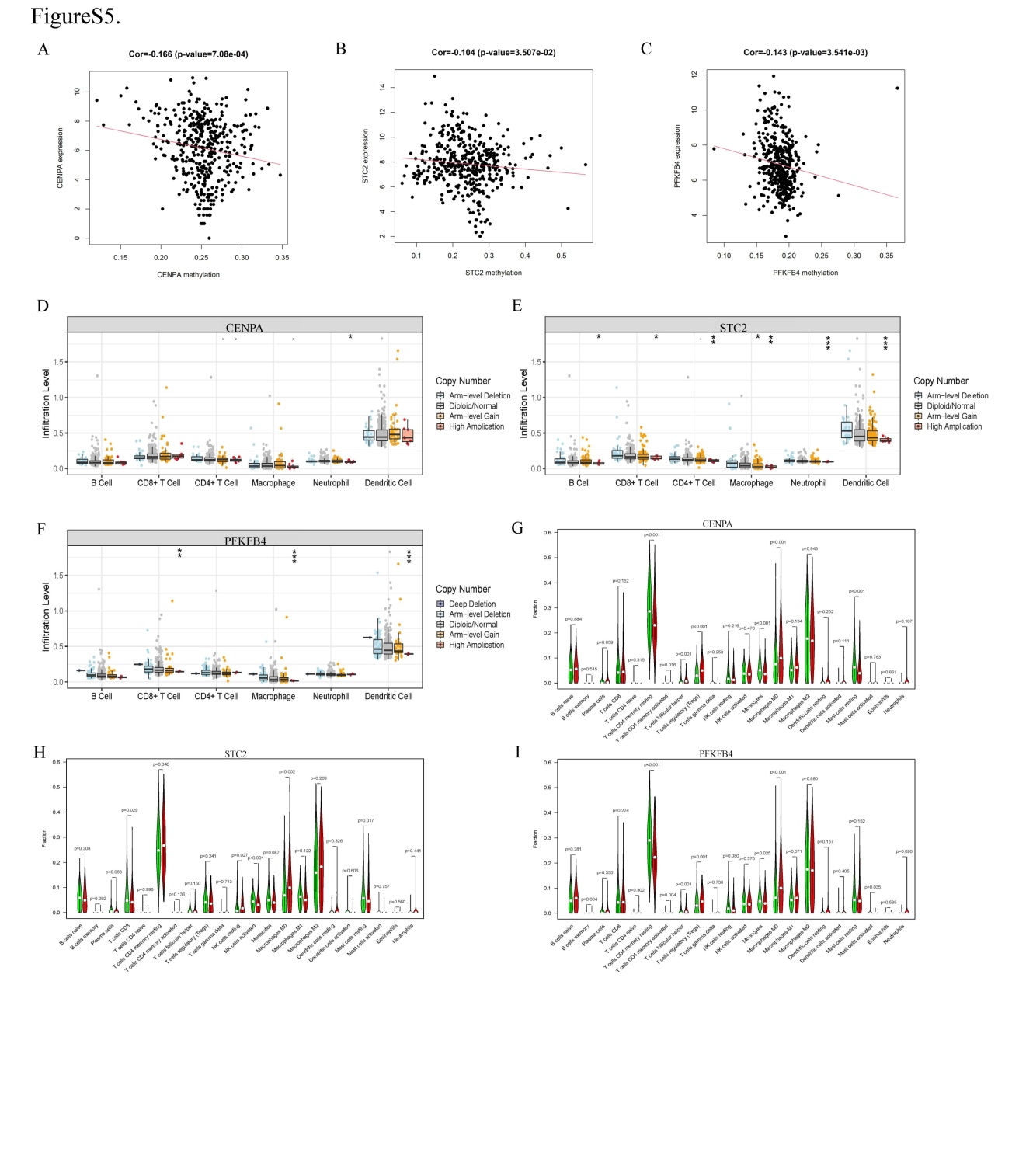


Figure S5. Molecular and immune characteristics of hub genes in the glycometabolism-related risk score. (A-C) Correlation between CENPA, STC2, and PFKFB4 gene expression and methylation level. (D-F) Differences in copy number variation of CENPA, STC2, and PFKFB4 in different immune cells. (G-I) Comparison of immune infiltrating cells between high and low expression groups of CENPA, STC2, and PFKFB4.
